# Listeriosis in Spain based on hospitalisation records, 1997 to 2015: need for greater awareness

**DOI:** 10.2807/1560-7917.ES.2019.24.21.1800271

**Published:** 2019-05-23

**Authors:** Zaida Herrador, Alin Gherasim, Rogelio López-Vélez, Agustín Benito

**Affiliations:** 1 National Centre for Tropical Medicine, Health Institute Carlos III (ISCIII in Spanish), Madrid, Spain; 2 Network Biomedical Research on Tropical Diseases (RICET in Spanish), Madrid, Spain; 3 National Centre of Epidemiology, Health Institute Carlos III (ISCIII in Spanish), Madrid, Spain; 4 Network Biomedical Research Centre in Epidemiology and Public Health (CIBERESP in Spanish), Madrid, Spain; 5 National Referral Unit for Tropical Diseases, Infectious Diseases Department, Ramón y Cajal Hospital, Instituto Ramón y Cajal de Investigación Sanitaria, Madrid, Spain

**Keywords:** Spain, food-borne infections, bacterial infections, listeriosis, Listeria, infection control, surveillance, clinic, epidemiology

## Abstract

**Introduction:**

Listeriosis is a food-borne disease of public health importance that has recently been involved in prolonged outbreaks. Despite its relevance, listeriosis is under-reported in many European countries.

**Aim:**

We aimed to describe listeriosis epidemiology in Spain from 1997–2015.

**Methods:**

We performed a retrospective study using the Spanish hospitalisation database. We calculated the mean number of hospitalisations per year and region. Pregnancy and neonatal-related listeriosis rates were computed. Relation between death and the presence of underlying health conditions was explored.

**Results:**

Between 1997–2015, 5,696 listeriosis hospitalisations occurred, showing a constantly increasing trend. Higher hospitalisation rates were located in the north of the country compared to southern regions. The age group ≥ 65 years old was the most represented (50%). Pregnant women and newborns accounted for 7% and 4% of hospitalisations, respectively. An underlying immunocompromising condition was present in 56.4% of patients: cancer (22.8%), diabetes mellitus (16.6%) and chronic liver disease (13.1%). Death occurred in 17% of patients, more frequently among those ≥ 65 years old (67.5%), with sepsis (39.9%) or with meningoencephalitis (19.2%).

**Conclusion:**

Listeriosis is an emergent public health problem in Spain that calls for targeted action. Further prevention strategies are urgently needed, including food safety education and messaging for all at-risk groups.

## Introduction

Listeriosis is an infectious disease caused by bacteria of the genus *Listeria* spp. *L. monocytogenes* is the major pathogenic species in both animals and humans. *L. monocytogenes* is a Gram-positive, rod-shaped organism that can grow in aerobic and anaerobic conditions [1], is widely distributed in the environment and is able to contaminate a wide variety of foods or beverages (soft cheese, deli meats, unpasteurised milk, refrigerated smoked seafood, etc.) [2]. The bacteria can multiply at refrigerator temperatures [3]; therefore, contaminated products are often kept for several days or even weeks, e.g. in the household/restaurants, and may be eaten on multiple occasions, which can complicate the identification of the incriminated food source [4].

The clinical syndromes of listeriosis include: febrile gastroenteritis, sepsis, central nervous system (CNS) involvement in the form of encephalitis, meningoencephalitis and focal infections such as pneumonia myo-endocarditis and septic arthritis, etc [5]. Invasive listeriosis most commonly affects pregnant women, neonates, elderly people and people with chronic conditions or weakened immune response [6]. Listeriosis has one of the highest case fatality rates among all food-borne infections; when it affects the CNS, the mortality rate is above 50% and neurological sequelae are present in more than 60% of survivors [2]. Listeriosis is also associated with fetal and neonatal death.

Worldwide, listeriosis is an emerging infection of public health concern [7]. In Europe, human listeriosis peaked in incidence during the 1980s, showed a general decline during the 1990s and stabilised in the 2000s [8]. More recent data show an increasing trend since 2008 [9]. This increase seems to be related to the ageing of the population and the increase in life expectancy of immunocompromised patients, but also to changes in the ways food is produced, stored, distributed and consumed around the world [10]. Although listeriosis is often a sporadic disease [11], large food-borne outbreaks have occurred during the last decade in Europe and the United States (US) [12]. In South Africa, an outbreak with more than 1,024 laboratory-confirmed listeriosis cases, as at 2 May 2018, has been ongoing since the start of 2017, with a 28.6% case fatality rate [13].

In Spain, food safety criteria (FSC) for *L. monocytogenes* follow European Commission (EC) regulations [14,15]. Before 2015, when it was added to the list of mandatory notifiable diseases, regions could voluntarily report listeriosis to the Microbiological Information System (Sistema de Información Microbiológica, SIM) [16]. Using the centralised hospital discharge database (Conjunto Mínimo Básico de Datos, CMBD), we aimed to describe the epidemiology of listeriosis in Spain from 1997–2015.

## Methods

### Data analysis

Using the CMBD database, we performed a retrospective descriptive study of listeriosis epidemiology between 1 January 1997–31 December 2015. The CMBD database receives notifications from around 98% of the public hospitals in Spain [17]. The National Health System (NHS) provides free medical care to 99.5% of the Spanish population; however, those who are not covered by the NHS can also be treated at public hospitals [18]. For this study, the International Classification of Diseases, Ninth Revision, Clinical Modification (ICD-9-CM) was used [19]. The case definition was a patient with ICD-9-CM diagnostic listeriosis (ICD-9 code: 027.0), placed in any diagnostic position. Relevant underlying conditions were also explored by searching for all related conditions in any diagnostic position. According to the literature, some of these conditions are malignant neoplasm (ICD-9 code: 140–209), diabetes mellitus (ICD-9 code: 250), chronic liver disease (ICD-9 code: 571), HIV infection (ICD-9 code: 042) and other immunosuppressive condition (ICD-9 code: 279).

In order to assess temporal and geographical patterns, the average number of hospitalisations per year and region (Comunidad Autónoma, CC.AA) were calculated. The population figures from the National Statistics Institute (Instituto Nacional de Estadística, INE) website were used to determine the population at risk [20]. Mortality data and live birth statistics, available since 1998, were also obtained from the INE website. To estimate the number of pregnant women, the following previously published method was applied: the fertility rate was multiplied by nine-twelfths of the population of women of reproductive age, since pregnancy lasts an average of 9 months. Similarly, the abortion/miscarriage rate was multiplied by a sixth of the population of women of reproductive age, since abortion usually occurs after 2 months of pregnancy. These two numbers were added to estimate the number of women who were pregnant per year during the study period [21].

Hospitalisation trends were assessed by age group and region. Results in terms of mean rates by CC.AA were plotted on maps using the Geographical Information System QGis free software version 2.18.13 (QGIS Development Team).

Socio-demographic and clinical data were extracted from the CMBD. Age was categorised in four groups: ≤ 15, 16–44, 45–64 and ≥ 65 years old, to provide an overview of children, young adults, older adults and elderly people. Hospitalisation costs were calculated using diagnostic cost groups based on diagnosis-related groups (DRGs) for hospitalised patients and their age, sex and resource consumption. DRGs represent a medical-economic entity, i.e. a set of diseases requiring analogous management resources [22].

Frequencies and percentages were used to summarise data. The association between related health conditions and death was assessed using a chi-squared test. Logistic regression models were obtained using a manual backward stepwise procedure and risk ratios (RRs) were computed with 95% confidence intervals (CI). Age was included as an adjustment variable and p values < 0.05 were considered statistically significant. Data analysis was performed using STATA software version 14 (StataCorp LLC, College Station, US).

### Ethical statement

This study involves the use of patient medical data from the CMBD. These data are hosted by the Ministry of Health, Consumer Affairs and Social Welfare (MSCBS). Researchers working in public and private institutions can request access to the database by filling in and signing a questionnaire available on the MSCBS website. In this questionnaire, a signed confidentiality commitment is required. All data are anonymised and de-identified by the MSCBS before it is made available to applicants [17].

## Results

### Spatial and temporal trends in Spain

Between 1997–2015, there were 5,696 hospitalisations with diagnosis of listeriosis in any diagnostic position recorded in the CMBD database. The ICD-9 code for listeriosis was positioned as first diagnosis in 3,110 (54.6%) of all such hospitalisations and as second diagnosis in 1,802 (31.6%). The mean listeriosis hospitalisation rate for the study period was 0.67 per 100,000 population (range: 0.19 in 1997 to 1.01 in 2013), with an increasing trend over the study period (p = 0.017).

**Figure 1:**
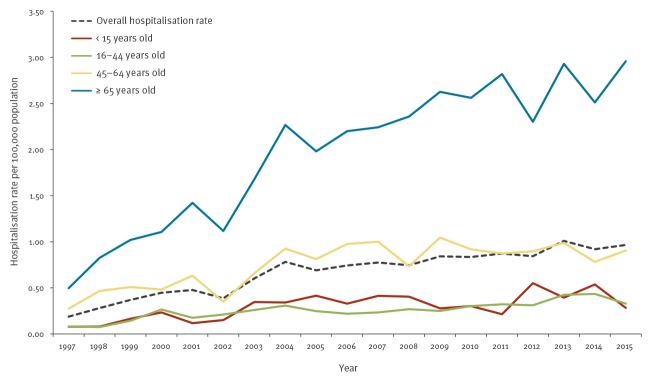
Listeriosis hospitalisation rate by age group, Spain, 1997–2015 (n = 5,696)

When comparing listeriosis hospitalisation rates by age group, relevant and significant differences emerged (p < 0.05). The age group ≥ 65 years old showed the highest rate, followed by 45–64 year olds. For those ≥ 65 years old, the mean hospitalisation rate in 2004–15 was more than twice that of the previous period: 2.48 vs 1.09 per 100,000 population, respectively (Figure 1).

**Figure 2:**
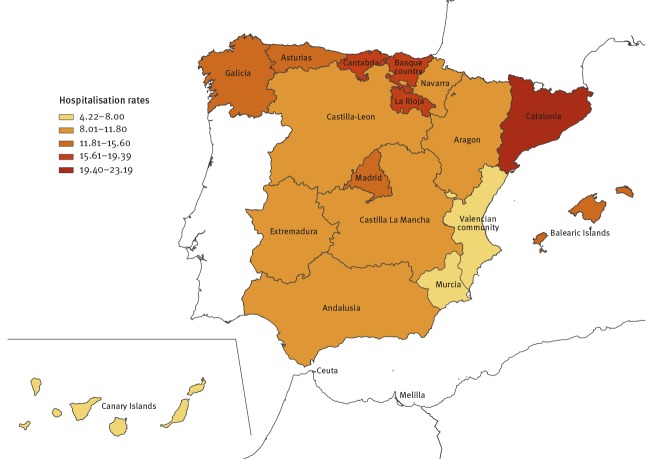
Listeriosis hospitalisation incidence rates per 100,000 population, by region, Spain, 1997–2015 (n = 5,696)

Regarding the regional distribution, most CC.AA with higher hospitalisation rates were located in the north of the country. Catalonia had the highest incidence rates of listeriosis hospitalisations (23.19 hospitalisations/100,000 population), followed by Cantabria (18.87/100,000 population), Rioja (17.09/100,000 population), Basque country (17.03/100,000 population) and Galicia (14.82/100,000 population) (Figure 2).

### Socio-demographic characteristics and clinical features of listeriosis-related hospitalisations

**Figure 3:**
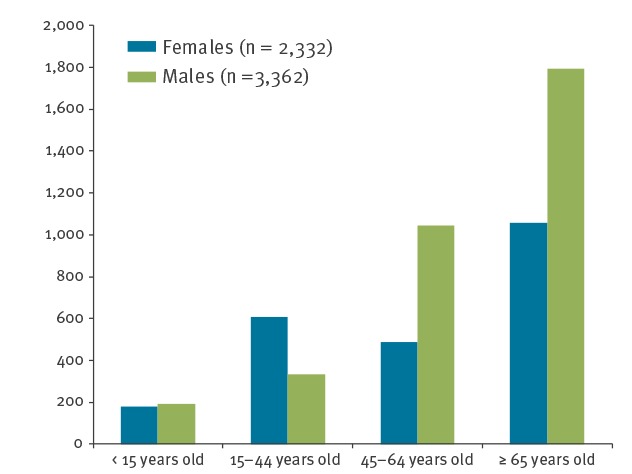
Number of listeriosis hospitalisations by sex and age group, Spain, 1997–2015 (n = 5,694)

The mean age of the 5,696 listeriosis-hospitalised patients was 58.6 years, with a median value of 64.5 years (Interquartile range (IQR): 47–75). Of those hospitalised, 59% were male (Table 1). The median age was 66 years (IQR: 53–75) in men and 61 years (IQR: 35–76) in women. As shown in Figure 1, the age group ≥ 65 years old was the most represented (50%). Males predominated in the age groups 45–64 years old (68.1%) and ≥ 65 years old (63%), while females represented 64.6% of those 15–44 years old (Figure 3; p < 0.01).

**Table 1:** Socio-demographic characteristics of listeriosis hospitalisations, Spain, 1997–2015 (n  =  5,696)

Variables		N	%
Sex	Male	3,362	59.0
	Female	2,332	41.0
Age group (years)	≤ 15	373	6.5
	15–44	943	16.6
	45–64	1,532	26.9
	≥ 65	2,848	50.0
Type of admission	Urgent	5,212	91.7
	Programmed	469	8.3
Type of discharge	Home	4,245	75.4
	Transfer	420	7.4
	Death	968	17.2
**Variables**	**Mean**	**Median**	**IQR**
Age (years)	58.6	64.5	47–75
Hospitalisation length of stay (days)	21.4	17	9–26
Hospitalisation cost (Euros)	38,157	6,327	4,680–6,858

IQR: interquartile range.

Certain parallels were observed between the increase in the number of people ≥ 65 years old who were hospitalised for listeriosis and the increase in life expectancy in Spain during the same period (Supplementary Figure 1).

The average length of hospital stay for listeriosis was 21.4 days. We found a wide range for the hospitalisation costs, with a median value of EUR 6,327 per case (IQR:  4,680–6,858), with no significant changes over time (Table 1).

Meningoencephalitis and/or septicaemia was seen in 43.3% and 8% of listeriosis hospitalisations, respectively. Males developed meningoencephalitis more often than women (47.4% vs 37.3%; p < 0.01). No differences were observed between males and females for septicaemia. Meningoencephalitis was more frequent among the age group 45–64 years old (50.1%), while those  ≥ 65 years old were more likely to have developed septicaemia (9.2%; p < 0.01).

Fatal outcome occurred in 17% of all listeriosis hospitalisations. The mean mortality rate was 0.06 per 100,000 population. Patients aged ≥ 65 years old accounted for 67.5% of deaths (p < 0.001). Fatal outcome happened in 19.2% of meningoencephalitis (RR: 1.26; 95% CI: 1.12–1.41; p < 0.01) and 39.9% of patients with septicaemia (RR: 2.66; 95% CI: 2.34–3.03; p < 0.01).

### Pregnancy-related listeriosis and neonatal listeriosis

**Figure 4:**
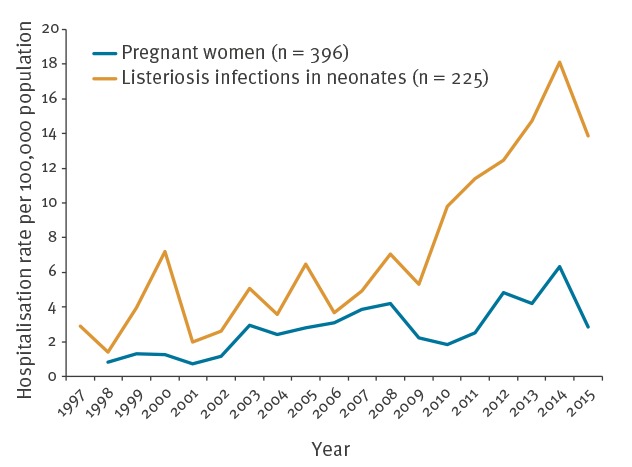
Listeriosis hospitalisation rates in pregnant women and neonates, per 100,000 live births, Spain, 1998–2015 (n = 621)

Pregnant women accounted for 7% (n = 396) of listeriosis hospitalisations. The mean infection rate during pregnancy was 7.19 per 100,000 pregnant women, showing several peaks (Figure 4) and a significant increase during the study period. No fatal outcomes were registered among pregnant women (p < 0.001).

Neonatal infections represented 4% (n = 225) of the overall listeriosis hospitalisations. The mean neonatal infection rate for the study period was 2.75 per 100,000 live births, indicating an increase from 1998–2007 (no cases were registered in 1997). This was followed by a steady decline in 2008–09, an increase from 2010–14 and finally a steady decline from 2015. Fatal outcome was less frequent in infected neonates than in the overall population (8.9% vs 17.3%; p < 0.01).

### Immunodeficiency-related listeriosis

Underlying immunocompromising conditions were observed in 56.4% (3,213/5,696) of the hospitalised patients: malignant neoplasm (n = 1,298; 22.8%), diabetes (n = 943; 16.6%), chronic liver disease (n = 747; 13.1%), HIV infection (n = 151; 2.7%) and other immunocompromising conditions (n = 69; 1.2%). Within the malignant neoplasm group, particularly frequent diagnoses were secondary malignant neoplasm (n = 480; 8.4%; ICD-9: 196–198), malignant neoplasm of trachea bronchus and lung (n = 164; 2.9%; ICD-9: 162), lymphoid leukaemia (n = 146; 2.6%; ICD-9: 204), other malignant neoplasms of lymphoid and histiocytic tissue (n = 132; 2.3%; ICD-9: 202), malignant neoplasm of colon (n = 94; 1.7%; ICD-9: 153) and malignant neoplasm of liver and intrahepatic bile ducts (n = 67; 1.3%; ICD-9: 155).

Hospitalised patients aged ≥ 65 years old were more likely to have malignant neoplasm and/or diabetes mellitus (p < 0.01), while chronic liver disease was more frequent in patients 45–64 years old (45.9%; p < 0.01). HIV-related listeriosis in 15–44 year old patients accounted for 55% of the listeriosis-hospitalised patients with HIV (p < 0.01).

Relation between fatal outcome and the presence of an underlying health condition was explored and adjusted by age. Fatal outcome occurred more frequently in hospitalised patients with listeriosis and co-diagnosis of diabetes mellitus (adjusted RR: 1.33; 95% CI: 1.10–1.60) and/or malignant neoplasm (aRR: 1.90; 95% CI: 1.63–2.22). Among the most prevalent malignant neoplasms, only secondary malignant neoplasm and malignant neoplasm of trachea, bronchus and lung increased the risk of death (Table 2).

**Table 2:** Related health conditions and fatal outcome in listeriosis hospitalisations, Spain, 1997–2015 (n = 5,696)

Related health conditions			Death				RR	95% CI	aRR	95% CI
			No		Yes					
			n	%	n	%				
Malignant neoplasm	All	Yes	950	73.19	348	26.81	1.90	1.69–2.13*	1.90	1.63–2.22*
		No	3,778	85.90	620	14.10				
	Secondary malignant neoplasm	Yes	317	66.04	163	33.96	2.20	1.91–2.53*	2.61	2.12–3.21*
		No	4,411	84.57	805	15.43				
	Malignant neoplasm of trachea bronchus and lung	Yes	104	63.41	60	36.59	2.22	1.81–2.75*	2.59	1.86–3.60*
		No	4,624	83.59	908	16.41				
	Lymphoid leukaemia	Yes	121	82.88	25	17.12	1.00	0.70–1.45	0.85	0.55–1.32
		No	4,607	83.01	943	16.99				
	0ther malignant neoplasms of lymphoid and histiocytic tissue	Yes	100	75.76	32	24.24	1.44	1.06–1.96*	1.36	0.90–2.05
		No	4,628	83.18	936	16.82				
	Malignant neoplasm of colon	Yes	73	77.66	21	22.34	1.32	0.90–1.94	1.12	0.69–1.84
		No	4,655	83.10	947	16.90				
	Malignant neoplasm of liver and intrahepatic bile ducts	Yes	49	73.13	18	26.87	1.59	1.07–2.37*	1.43	0.83–2.48
		No	4,679	83.12	950	16.88				
HIV infection		Yes	129	85.43	22	14.57	0.85	0.58–1.26	1.49	0.93–2.41**
		No	4,599	82.94	946	17.06				
Diabetes mellitus		Yes	781	82.82	162	17.18	1.01	0.87–1.18	1.33	1.10–1.60*
		No	3,947	83.04	806	16.96				
Chronic liver diseases		Yes	603	80.72	144	19.28	1.16	0.99–1.36**	1.13	0.92–1.38
		No	4,125	83.35	824	16.65				

aRR: adjusted risk ratio; CI: confidence interval; RR: relative risk.

* p < 0.05; **p < 0.10.

## Discussion

This study provides a 19-year review of listeriosis hospitalisations in Spain. To date, the few articles that have been published on listeriosis epidemiology in Spain were focused on outbreak investigations or based on incomplete information sources [23-25]. To our knowledge, this is the first study to address listeriosis epidemiology nationwide.

Between 1997–2015, a total of 5,696 listeriosis-related hospitalisations occurred in Spain. From 2009–13, there were 588 listeriosis cases reported to SIM, the European Surveillance System (TESSy) and the corresponding yearly European Food Safety Authority (EFSA) reports [26]; however, 2,051 listeriosis-related hospitalisations were recorded in the CMBD, indicating considerable under-reporting. Even if this indicates that the epidemiological scenario is quite uncertain, in 2012 the Spanish listeriosis rate reported to EFSA was the second highest of any country in the European Union (EU) (0.93, vs an EU-wide incidence rate of 0.41/100,000 population) [27]; this indicates that more attention needs to be given to preventing and controlling this disease in Spain.

Our results suggest an increasing trend of listeriosis infection in Spain during the study period (1997–2015). In the last decade, listeriosis incidence rates have increased or remained stable at relatively high levels across Europe. Despite the application of the FSC for *L. monocytogenes* in ready-to-eat (RTE) foods from 2006 onwards, a statistically significant increasing trend of human invasive listeriosis has been reported in the EU/European Economic Area (EEA) from 2009–13 [28]. From 2012–16, between 1,754 and 2,555 *L. monocytogenes* cases were reported annually to TESSy by 30 EU/EEA countries [3]. In the absence of systematic and national surveillance in several European countries, under-reporting may be masking an even worse scenario. In Spain, listeriosis became a mandatory notifiable disease in 2015 [29]. As demonstrated in the Netherlands, public health investment in surveillance can yield an increase in reported cases of listeriosis [30]; therefore, an increase in listeriosis detection may be expected in the future.

The increasing proportion of susceptible persons in the general population is a feasible contributing factor to this increased incidence [31]. In our study, listeriosis rates increased mostly in those ≥ 65 years old; according to the 2015 EFSA report, the proportion of cases in this age group steadily increased from 56% in 2008 to 64% in 2015 [9], which is similar to our results.

Another potential contributing factor to the overall increase in listeriosis is the rise in consumption of RTE foods. European surveys have revealed associations between listeriosis and several parameters, including food packaging types, preparation practices and storage temperatures; the stage of sampling with respect to shelf life; and a lack of education and training of food handlers [11].

The emergence of particularly virulent strains of *Listeria* spp. is also of concern for public health. It has been demonstrated that *L. monocytogenes* can exhibit tolerance to quaternary ammonium disinfectants, as well as temperature-dependent resistance to phages, which can be gained through acquisition of new genes as well as mutations in existing genes [32] Moreover, it seems that differences among strains have an impact on virulence of specific immunocompromised subpopulations [8]. Another possible explanation is the increased consumption of drugs that reduce gastric acidity. In humans, the low pH of the stomach provides a significant barrier to *Listeria* spp. infection and it has been demonstrated that patients taking medications that reduce gastric acid (like proton pump inhibitors (PPI)) are at increased risk of infection [33]. In 2010, PPI became the most commonly consumed active compound in Spain, in terms of number of packages sold. In fact, in comparison to other European countries, Spain leads gastric anti-secretory drug consumption with the number of prescriptions soaring 70% over the European average [34].

During the study period, only a few listeriosis outbreaks in Spain were reported in the literature, mostly in the Basque country. These outbreaks were related to the consumption of Latin-style fresh cheese made from pasteurised milk in Portugal [35] and cooked ham [36]. In both situations, health professionals were informed and were recommended to strongly consider the diagnosis of listeriosis in high-risk individuals. Genetic studies also revealed the occurrence of an outbreak in Castilla-Leon, which was unreported and thus the source of the outbreak was not traced [37]. Several outbreaks due to the consumption of ‘vegetables and juices and other products thereof’ were also reported to EFSA during 2010–16 [3]. Furthermore, the prevalence of *L. monocytogenes* in RTE products in markets in northern Spain was recently studied, finding that smoked fish was the most frequently contaminated food category [38]. These findings and the outbreaks mentioned above influenced Spain’s change to mandatory national reporting of listeriosis cases in 2015 [16]. The Spanish Agency for Consumer Affairs, Food Safety and Nutrition (Agencia Española de Seguridad Alimentaria y Nutrición) receives several notifications every year and informs the regional authorities via the national Food Alert Network and the European Commission Services via the Rapid Alert System for Food and Feed; at the national level, contaminated products are recalled from markets [39]. It is nevertheless possible that additional outbreaks and/or clusters may have gone unnoticed due to the lack of surveillance.

Improvements in listeriosis surveillance (including accurate microbiological investigations) with active collaboration between public health officials and food regulatory officials should improve the situation. Data sharing at the European level is also essential, given that many food-borne disease outbreaks happen in a multi-country setting [3]. Without further action, yearly numbers are likely to continue to grow, for the aforementioned reasons.

We found relevant disparities between regions, with higher hospitalisation rates mostly in the north of the country. This might be due to differences in dietary habits, food consumption or regulations; the population’s average age; differences in educational preventive measures and/or that health professionals are more prone to seek listeriosis diagnosis. However, further investigation is needed to confirm these hypotheses.

In this study, we found that from 1997–2015 *Listeria* spp. infection in Spain was more common among males. The origin of the sex preference of *L. monocytogenes* infection has not yet been clarified, but it seems to be related to susceptibility to infection, as demonstrated for other infectious diseases [40]. The highest hospitalisation rate for both sexes was seen in the ≥ 65 age group, with a higher rate for males than for females. However, females accounted for more hospitalisations in the age group 15–44, which is believed to largely reflect pregnancy‐related listeriosis. The mean length of hospital stay and associated costs, including for those individuals who ultimately died as a result of their infections, were quite high, close to those in an outbreak in Canada [41]. In a study carried out in the Netherlands, perinatal listeriosis accounted for the highest cost-of-illness per infected case among the 14 most frequent food-related pathogens [42].

Death occurred in 17% of the listeriosis hospitalisations observed in this study. In the literature, the overall listeriosis mortality rate ranged from 20–30% [43], a bit higher than the rate we encountered. Considering that we only analysed hospitalised patients, the opposite should have been expected. Nevertheless, non-invasive listeriosis cases were also identified. In fact, fatal outcome in listeriosis hospitalisations was significantly associated with being ≥ 65 years old, the presence of meningoencephalitis or septicaemia, and some underlying conditions.

An increasing trend in listeriosis hospitalisation rate was observed in pregnant women. According to a recent EFSA report, listeriosis cases in Europe have increased among women aged 25–44. It is believed that these cases are mainly pregnancy related [28]. In the US and France, the incidence of listeriosis in pregnant women declined in the 1990s after the implementation of industrial, regulatory and educational measures. Moreover, the incidence of pregnancy-related listeriosis was lower in French regions with a low prevalence of toxoplasmosis [44], which may be related to differences in educational measures to prevent both diseases. In Spain, there is currently no screening for listeriosis during pregnancy, nor specific dietary recommendations for pregnant women [45]; therefore, dietary recommendations and screening activities during pregnancy should be introduced.

Malignant neoplasm, mostly hematologic disease, was observed in 22.8% of listeriosis-related hospitalisations. Cancer and immunosuppressive therapy were among the most frequently recorded comorbid conditions in non-pregnancy–associated cases of listeriosis, mainly due to impaired cell-mediated immunity and other underlying conditions [5]. Moreover, the mortality rate of *L. monocytogenes* infection is elevated in cancer patients, as infections can be more difficult and time consuming to treat [46]. Secondary malignant neoplasm and malignant neoplasm of trachea, bronchus and lung were the malignant neoplasms with the highest probability of fatal outcome. In a review of listeriosis cases reported in France from 2001–08, patients with lung and pancreatic cancer had the highest case fatality rate of listeriosis [47].

Fatal outcome also occurred more frequently in hospitalised patients with chronic liver diseases and/or diabetes mellitus, although statistical difference only remained significant for diabetes mellitus after adjustment by age. According to a review of listeriosis cases worldwide, patients with cancer and type 2 diabetes mellitus have five times the risk of contracting listeriosis [48]. Diabetes mellitus may lead to an immunocompromised state by decreased efficacy of cell-mediated immunity; for example, diabetic patients are twice as susceptible to bacterial meningitis as patients who are not diabetic [49]. Moreover, patients with diabetes may have additional immunocompromising conditions. Health professionals should be aware of this and specific food recommendations should be given to these patients, together with other public health general interventions.

### Limitations and conclusions

Our study has several limitations. Even if the CMBD provide information from a network of hospitals that covers more than 99% of the population in Spain [13], hospital discharge records do not include cases managed in an outpatient setting or asymptomatic cases; thus, hospital records underestimate the real burden of listeriosis in Spain. Moreover, the CMBD does not provide information about the laboratory tests used for listeriosis diagnosis, which impairs the quality of the data. Also, we could not identify mother-baby pairs, as the personal information necessary to do this is missing from this database due to data protection.

In conclusion, our report highlights that listeriosis is an important public health problem in Spain that needs to be prioritised, especially due to its increasing trend and severity. Listeriosis surveillance needs to be improved and further targeted prevention is urgently needed, including food safety education and messaging in all at-risk groups. Implementing, for example, the US Centre for Disease Control’s recommendations for the prevention of listeriosis for the general public—especially high-risk populations—may be a starting point [25]. Furthermore, industrial and regulatory measures needs to be implemented in parallel, as an integrated and multi-sectoral approach is the only way to successfully prevent and control listeriosis.
